# Twist1 Promotes Gastric Cancer Cell Proliferation through Up-Regulation of FoxM1

**DOI:** 10.1371/journal.pone.0077625

**Published:** 2013-10-24

**Authors:** Jianxin Qian, Yizhou Luo, Xiaoqiang Gu, Wang Zhan, Xi Wang

**Affiliations:** 1 Department of Medical Oncology, Changzheng Hospital, Shanghai, China; 2 Department of Oncology & Hematology, the 454th Hospital of PLA, Nanjing, China; Virginia Commonwealth University, United States of America

## Abstract

Twist-related protein 1 (Twist1), also known as class A basic helix-loop-helix protein 38 (bHLHa38), has been implicated in cell lineage determination and differentiation. Previous studies demonstrate that Twist1 expression is up-regulated in gastric cancer with poor clinical outcomes. Besides, Twist1 is suggested to be involved in progression of human gastric cancer. However, its biological functions remain largely unexplored. In the present study, we show that Twist 1 overexpression leads to a significant up-regulation of FoxM1, which plays a key role in cell cycle progression in gastric cancer cells. In contrast, knockdown of Twist 1 reduces FoxM1 expression, suggesting that FoxM1 might be a direct transcriptional target of Twist 1. At the molecular level, we further reveal that Twist 1 could bind to the promoter region of FoxM1, and subsequently recruit p300 to induce FoxM1 mRNA transcription. Therefore, our results uncover a previous unknown Twist 1/FoxM1 regulatory pathway, which may help to understand the mechanisms of gastric cancer proliferation.

## Introduction

Epithelial-mesenchymal-transition (EMT) is a process whereby epithelial cells lose polarity and cell-to-cell adhesion, and undergo dramatic remodeling of the cytoskeleton [1,2]. Concurrent with loss of epithelial cell adhesion and cytoskeletal components, cells undergoing EMT acquire expression of mesenchymal components and a migratory phenotype [2,3]. 

Several key inducers of EMT are transcription factors including Twist 1, Snail, and Slug, which repress E-cadherin expression [4,5]. Twist1 belongs to the basic helix-loop-helix transcription factor family [6]. Initially, Twist1 was suggested to be essential in the development of mesodermally derived tissues, including muscle and osteogenic cell lineages [7,8]. Subsequent studies have shown that Twist 1 promotes EMT and plays an essential role in metastasis in several tumor models [9,10]. Expression of Twist 1 has also been implicated in promotion of metastasis and invasive pathological subtypes in several types of carcinoma [11]. Therefore, Twist 1 has been suggested to have oncogenic properties. For example, overexpression of Twist in rhabdomyosarcoma inhibits Myc-induced apoptosis and interferes with p53 tumor suppression [12]. Up-regulation of Twist is associated with malignant transformation in T-cell lymphoma [13]. Forced expression of Twist triggers resistance of human cancer cells to drugs that inhibit microtubule formation [14]. 

However, the effect and mechanism of Twist gene on proliferation of gastric carcinoma remain enigmatic. Recent studies have shown that Twist1 Is up-regulated in gastric cancer-associated fibroblasts with poor clinical outcomes [15]. Besides, down-regulation of the Twist 1 gene suppressed the proliferation of gastric cancer cells by negatively regulating the AP-1 activity resulting in the cyclin D1 expression decreasing [16]. In the present work, two gastric cancer cell lines were employed to investigate the effect and mechanism of Twist 1 gene on cell proliferation.

## Materials and Methods

### Cell Culture

Four epithelial cell lines (NCI-N87, AGS, HGC-27 and MGC80-3) derived from gastric carcinoma were obtained from American Type Culture Collection (USA). Cells were cultured in DMEM/F12 (Invitrogen, Carlsbad, CA) supplemented with 10% fetal bovine serum (Gibco, Beijing). Cultures were maintained at 37°C in a humidified atmosphere with 5% CO_2_.

### Small Interfering RNA, RNA extraction and Real-time Analysis

Cells were seeded on to 6-well plates then transfected with 50nM siRNA oligos targeting human Twist 1 (Dharmacon, USA). The siRNA molecule specific for green fluorescent protein (GFP) was used as negative control. Total RNAs were extracted from cells by TRIzol reagent, and reverse transcriptions were performed by Takara RNA PCR kit (Takara, China) following the manufacturer’s protocol. In order to quantify the transcripts of the interest genes, real-time PCR was performed using a SYBR Green Premix Ex Taq (Takara, Japan) on Light Cycler 480 (Roche, Switzerland). 

### Transient Transfections and Luciferase Assays

Human FOXM1 promoter was amplified from the human genomic DNA template and inserted into pGL4.15 basic vector (Promega). Mutant Twist1 binding motif was generated using a PCR mutagenesis kit (Toyobo) with a primer (mutation sites underlined): 5’-CGCATTACGAATCAGGTTAAGCCAGTT-3’ and a reverse complement primer. All the transient transfections were performed by Lipofectamine 2000 (Invitrogen, Shanghai), according to the manufacturer’s instructions. For the luciferase reporter assays, cells were seeded in 24-well plates and transfected with the indicated plasmids. 48 hours after transfection, luciferase activities were measured using the Dual Luciferase Reporter Assay System (Promega, USA).

### Co-immunoprecipitation

Cells were harvested, resuspended in lysis buffer containing 50 mM Tris-HCl (pH 7.3-7.5), 120 mM NaCl, 1 mM EDTA, 0.5% TRITON X-100) and protease inhibitors. Lysates were incubated with 2.5 µg of p300 or IgG overnight at 4°C. Protein A beads were added for additional 4 hours. Beads containing immune complexes were washed with 1 ml ice cold lysis buffer for four times. Precipitates were denatured in Laemmli (gel loading) buffer at 95°C for 10 min.

### Western Blot

Cells were harvested by trypsinization, lysed in Laemmli buffer, denatured for 10 min at 80°C, and resolved on SDS/PAGE gels. After immunoblotting, the membranes were blocked in PBS/0.1% Tween-20 with 7.5% nonfat dry milk, and primary antibodies were incubated in PBS/0.1% Tween-20 with 0.1%-5% nonfat dry milk. Antibodies directed against Twist 1, FoxM1 were purchased from Santa Cruz Biotechnology (USA). Anti-p300 and GAPDH antibodies were obtained from Abcam Company (USA). Anti-p21, p27, Cyclin B1, Cyclin D1, Cyclin D2, Cyclin E and E-cadherin antibodies were from Cellsignaling (USA).

### Chromatin Immunoprecipitation (ChIP) assay

Chromatin immunoprecipitation (ChIP) assay kits were used (Upstate, USA). Briefly, Cells were fixed with 1% formaldehyde and further processed using Upstate’s ChIP assay Kits. Soluble chromatin was immunoprecipitated with anti-Twist 1 and IgG antibodies. After purification, DNA samples were quantified by quantitative real-time PCR using primers encompassing proximal region of human FoxM1 promoter. 

### Statistical Analysis

All data are presented as mean±SEM. Statistical differences were determined by a two-tailed t test. Statistical significance is shown as * (P < 0.05), ** (P < 0.01) or *** (P < 0.001). 

## Results

### Effect of Twist1 on gastric cancer cell proliferation

**Figure 1:**
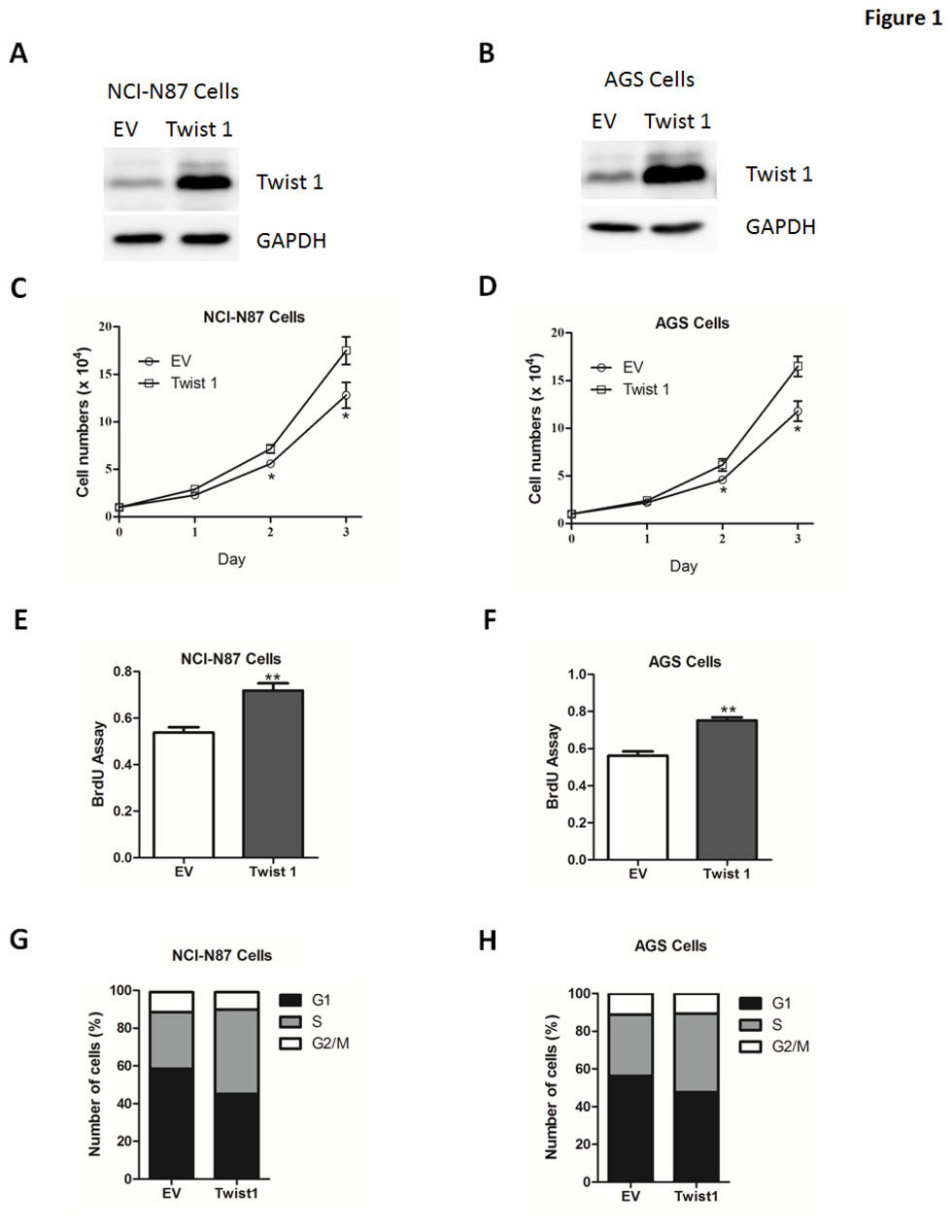
Twist 1 overexpression promotes gastric cell proliferation. (A-B) Representative western blot analysis of Twist 1 expression in NCI-N87 (A) or AGS (B) cells transfected with adenoviruses expressing empty vector (EV) or Twist 1. (C-D) The growth curve of NCI-N87 (C) or AGS (D) cells cells transfected with empty vector (EV) or Twist 1. (E-F) The cell proliferative potential (BrdU) was determined in NCI-N87 (E) or AGS (F) cells transfected with empty vector (EV) or Twist 1. (G-H) The cell cycle phase of NCI-N87 (G) and AGS (H) cells transfected with empty vector (EV) or Twist 1 was analyzed by flow cytometry. Cells were labeled for 15 min with PI and immediately analyzed by flow cytometry. Histograms represent the percentage of cells in each phase of the cell cycle (G0/G1, S and G2/M).

At the first, to explore the functional role of Twist 1 in cell proliferation, we transduced NCI-N87 or AGS cells with adenoviruses containing Twist 1 or empty vector (Figure 1A and 1B). Consistent with previous studies, the expression of E-cadherin, a biomarker of EMT process [4,5], was down-regulated by Twist 1 overexpression (Figure S1A-1B). Besides, Twist 1 overexpression resulted in a significant increase in cell number of all cells tested (Figure 1C and 1D). Consistently, bromodeoxyuridine (BrdU) analysis also confirmed that Twist 1 overexpression promoted cell proliferation (Figure 1E and 1F). Besides, Twist 1 overexpressing cells had a significantly lower percentage of cells in the G1/G0 phase and increased percentage of cells in the S phase, compared to empty vector-transfected cells (Figure 1G-1H).

**Figure 2:**
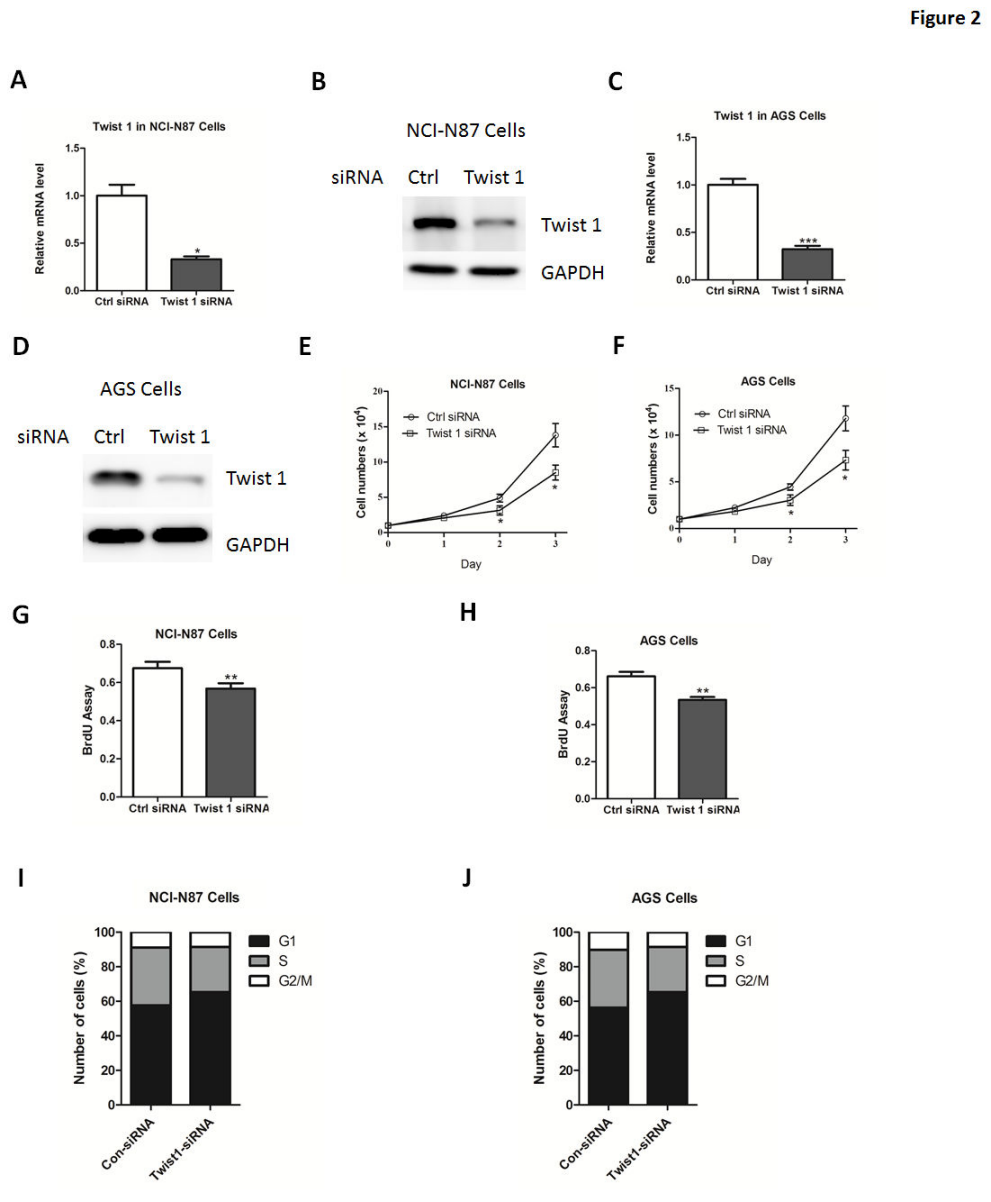
Twist 1 depletion inhibits gastric cell proliferation. (A-D) Quantitative real-time PCR and western blot analysis of Twist 1 expression in NCI-N87 (A-B) or AGS (C-D) cells transfected with siRNA oligos targeting Twist 1 or negative control siRNA (Ctrl). (E-F) The growth curve of NCI-N87 (E) or AGS (F) cells cells transfected with siRNA oligos targeting Twist 1 or negative control siRNA (Ctrl). (G-H) The cell proliferative potential (BrdU) was determined in NCI-N87 (G) or AGS (H) cells transfected with siRNA oligos targeting Twist 1 or negative control siRNA (Ctrl). (I-J) The cell cycle phase of NCI-N87 (I) and AGS (J) cells transfected with siRNA oligos targeting Twist 1 or scramble siRNA (Ctrl) were analyzed by flow cytometry.

Next, NCI-N87 or AGS cells were transfected with small interfering RNA (siRNA) targeting Twist 1. The siRNA oligo showed efficient Twist 1 knockdown in these two cells, compared with GFP siRNA-transduced cells (Figure 2A-2D). As a result, down-regulation of Twist 1 led to a marked decrease in cell number and proliferation in these cells (Figure 2E-2H). Additionally, silencing of Twist 1 significantly increased the percentage of cells in the G0/G1 phase and decreased the percentage of cells in the S phase (Figure 2I-2J). Notably, we compared the proliferation activity of NCI-N87 and AGS cells with or without transfection of empty vector or GFP siRNA. Our results suggest that neither empty vector or GFP siRNA affect the cell proliferation activity (Figure S2A). Besides, the growth ability of cells expressing higher Twist 1 levels (NCI-N87 and AGS cells) is relatively higher than others (HGC-27 and MGC80-3 cells) (Figure S2C). Taken together, our results suggest that Twist 1 might be an important positive regulation of gastric cancer cell proliferation.

### Twist1 affects expression of cell-cycle regulators

**Figure 3:**
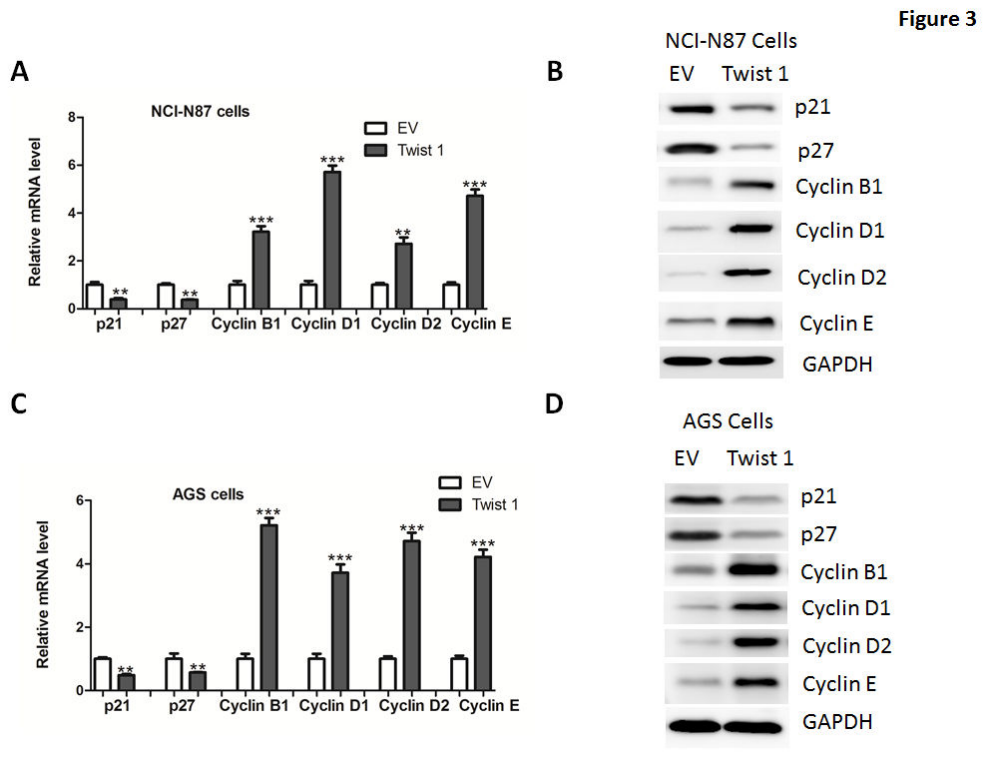
Twist 1 overexpression on the regulation of cell-cycle regulators. (A-D) Quantitative real-time PCR and western blot analysis of p21, p27, Cyclin B1, Cyclin D1, Cyclin D2 and Cyclin E in NCI-N87 (A-B) or AGS (C-D) cells transfected with adenoviruses expressing empty vector (EV) or Twist 1.

**Figure 4:**
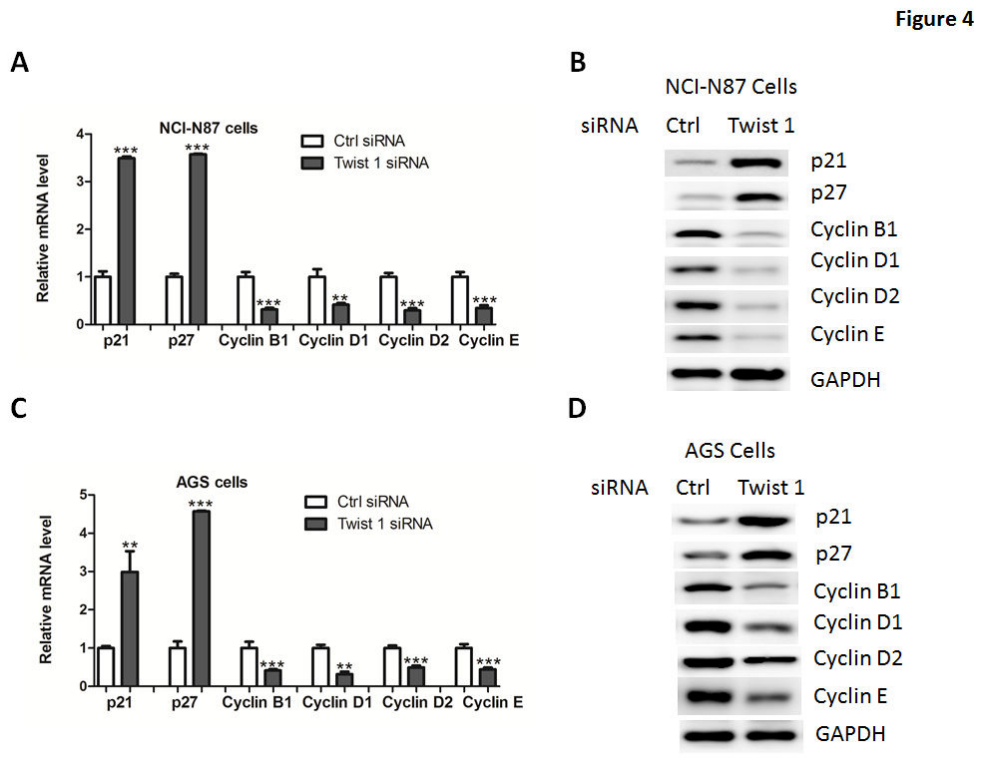
Twist 1 silencing on the regulation of cell-cycle regulators. (A-D) Quantitative real-time PCR and western blot analysis of p21, p27, Cyclin B1, Cyclin D1, Cyclin D2 and Cyclin E in NCI-N87 (A-B) or AGS (C-D) cells transfected with siRNA oligos targeting Twist 1 or negative control siRNA (Ctrl).

As Twist 1 promoted cell proliferation, we examined its functions on expression of the genes which regulate the G1/S transition, including the CDK inhibitors p21^Cip1^, p27^Kip1^, the CDK regulator Cyclin B1, Cyclin D1, Cyclin D2 and Cyclin E. Results from real-time PCR and western blotting analysis in NCI-N87 cells suggested that expression of p21^Cip1^, p27^Kip1^ were downregulated while cyclin B1, Cyclin D1, Cyclin D2 and Cyclin E levels were upregulated in Twist 1-transfected cells, compared to empty vector-transfected cells (Figure 3A-3B). Similar results were also observed in AGS cell (Figure 3C-3D), further confirming that Twist 1 can influence the proliferation of gastric cancer cells. Consistently, knockdown of Twist 1 increased p21^Cip1^ and p27^Kip1^ expression while reduced Cyclin B1, Cyclin D1, Cyclin D2 and Cyclin E expression in NCI-N87 and AGS cells (Figure 4A-4D).

### Twist 1 directly targets the transcription factor FoxM1 in gastric cancer cells

**Figure 5:**
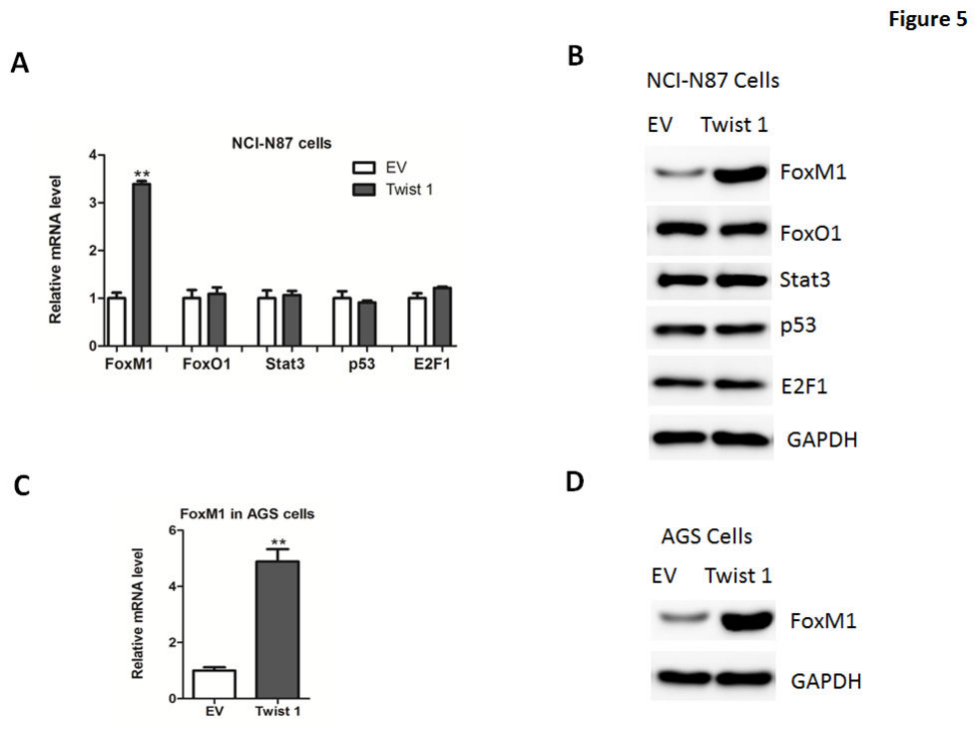
FoxM1 expression in gastric cancer cells overexpressing Twist 1. (A-B) Quantitative real-time PCR (A) and western blot (B) analysis of FoxM1, FoxO1, Stat3, p53 and E2F1 expression in NCI-N87 cells transfected with adenoviruses expressing empty vector (EV) or Twist 1. (C-D) Quantitative real-time PCR (C) and western blot (D) analysis of Twist 1 expression in NCI-N87 cells transfected with adenoviruses expressing empty vector (EV) or Twist 1.

**Figure 6:**
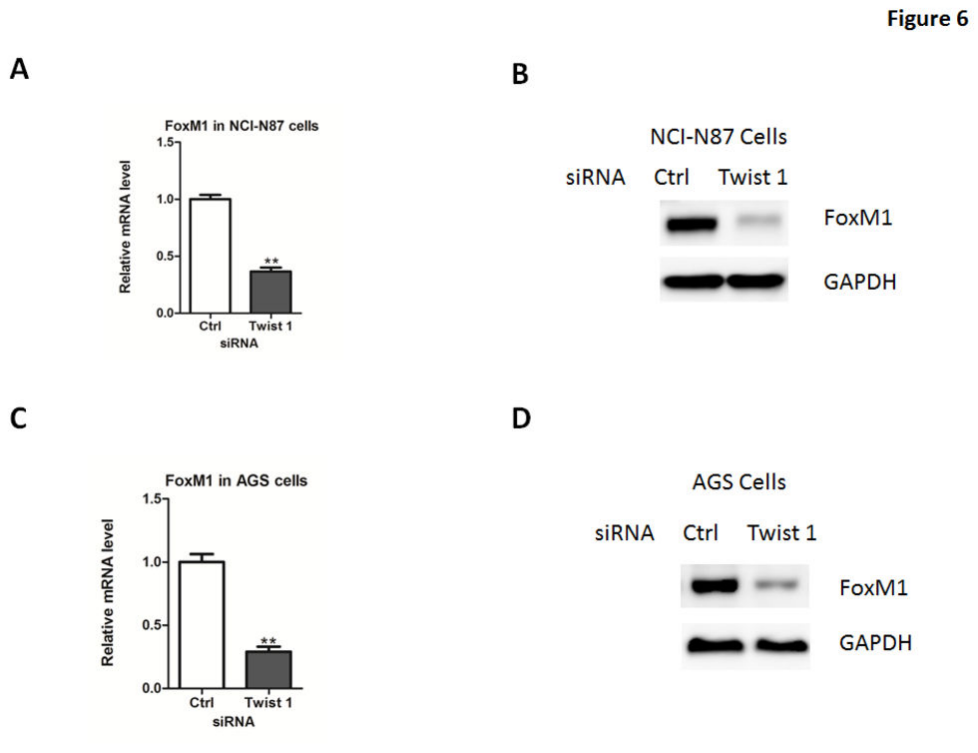
FoxM1 expression in gastric cancer cells depletion of Twist 1. (A-B) Quantitative real-time PCR (A) and western blot (B) analysis of FoxM1 expression in NCI-N87 cells transfected with siRNA oligos targeting Twist 1 or negative control siRNA (Ctrl). (C-D) Quantitative real-time PCR (C) and western blot (D) analysis of FoxM1 expression in NCI-N87 cells transfected with siRNA oligos targeting Twist 1 or negative control siRNA (Ctrl).

Previous studies have revealed that several transcriptional factors can regulate a series of genes relevant to the cell cycle, including p21^Cip1^, p27^Kip1^ and Cyclin B1. Thus, we analyzed the potential transcriptional factors that participate in regulation of cell proliferation. Out of five tested transcriptional factors, only FoxM1 were found to be significantly increased in NCI-N87 cells overexpressing Twist 1 (Figure 5A-5B). The induction of FoxM1 was also observed in AGS cells (Figure 5C-5D). In addition, FoxM1 expression was reduced in these cell depleted of Twist 1(Figure 6A-6D), suggesting that FoxM1 might be a target of Twist 1.

### Twist 1 upregulates FoxM1 expression through recruitment of p300

**Figure 7:**
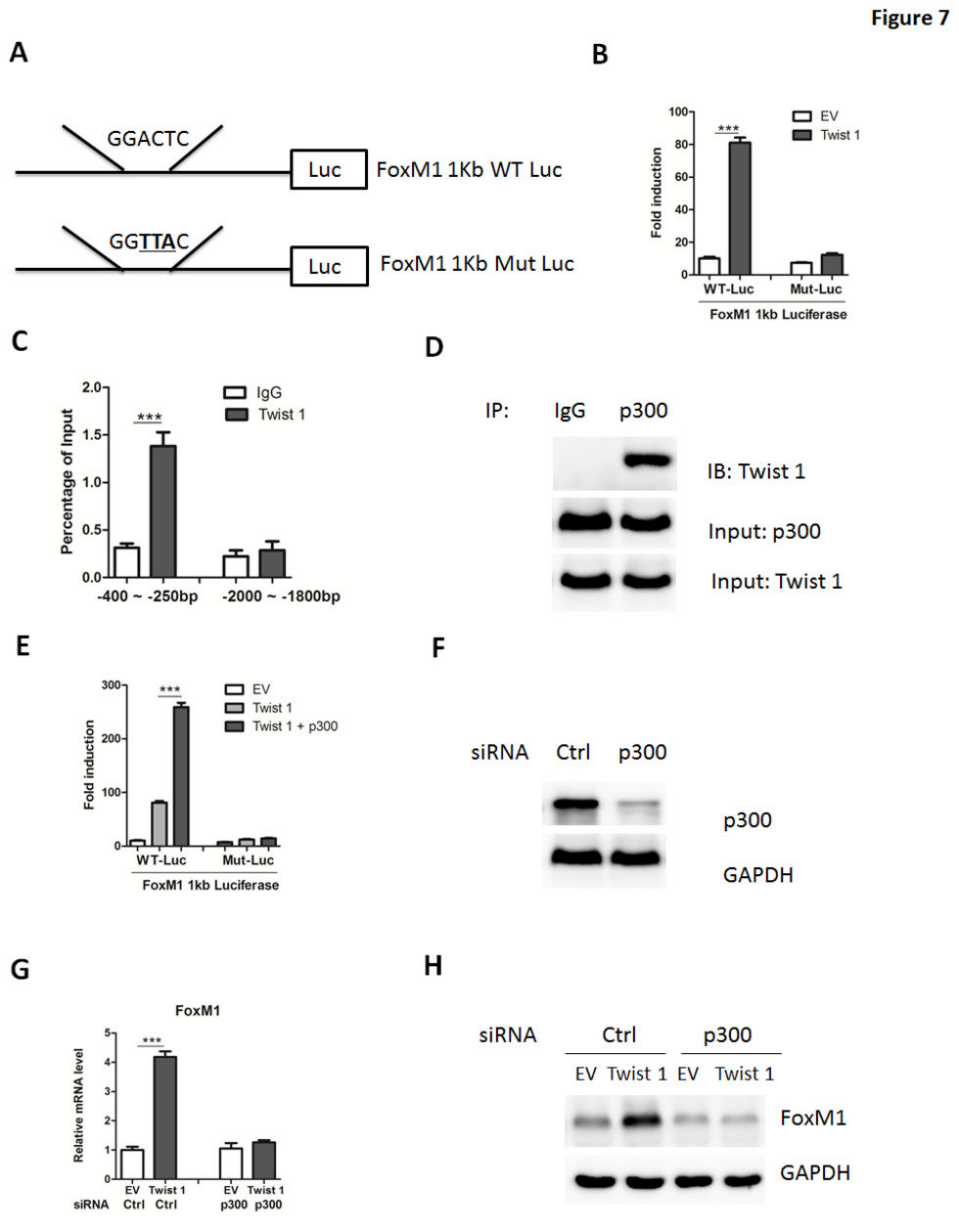
Twist 1 promotes FoxM1 promoter activity through recruiting p300. (A) Diagram of the Twist 1-binding site in the human FoxM1 promoter (1kb) and two FoxM1 luciferase reporters. WT-Luc: wild-type luciferase reporter; Mut-Luc: luciferase reporter carrying point mutations of Twist 1 binding site. Mutations were underlined. (B) Activation of FoxM1 luciferase reporters by Twist 1 in NCI-N87 cells. (C) The binding of Twist 1 on the promoter regions of human FoxM1 gene were analyzed by ChIP assays and quantified by real-time PCR. The region containing -2000 to -1800 bp were used as a negative control. (D) Co-immunoprecipitation of p300 and Twist 1 in NCI-N87 cells. (E) Activation of FoxM1 luciferase reporters by Twist 1 and p300 in NCI-N87 cells. (F) Representative western blot analysis of p300 expression in NCI-N87 cells transfected with siRNA targeting p300 or negative control (Ctrl). (G-H) Quantitative real-time PCR (G) and western blot (H) analysis of FoxM1 expression in NCI-N87 cells transfected with adenoviruses expressing empty vector (EV) or Twist 1. Cells were pre-transfected with siRNA oligos targeting p300 or negative control siRNA (Ctrl) for 24 hours.

Next, we focused on the molecular mechanism of Twist 1 regulation of FoxM1 transcription. Sequence analysis showed that the promoter region of human FoxM1 gene contained a potential Twist 1 binding site (between -357 and -352 bp) (Figure 7A). Luciferase report assay showed that the transcriptional activity of wild-type FoxM1 promoter was dramatically upregulated by Twist 1, whereas the transcriptional activity was abolished in the promoter bearing a mutation in Twist 1-binding sites (Figure 7B). Furthermore, ChIP assays showed that Twist 1 could uniquely bind to FoxM1 promoter (Figure 7C). To determine if induction of FoxM1 by Twist 1 is required for its proliferative effect, we carried out experiments with FoxM1 knockdown using siRNA oligos in NCI-N87 cells (Figure S3A-S3B). As a result, the siRNA rescued cells from the proliferative effect of Twist 1 overexpression (Figure S3C). Consistently, the functions of Twist 1 on the expression levels of cell-cycle regulators were also reversed by FoxM1 siRNA oligos (Figure S3D), suggesting that FoxM1 is required for the roles of Twist 1 in gastric cancer cells. 

As a transcriptional factor, Twist 1 can recruit coactivators in the regulation of target gene expression. Previous studies demonstrated that p300 could act as a coactivator for many transcription factors. We thus examined whether p300 could be a coactivator for Twist 1 regulation of FoxM1 expression. As shown in Figure 7D, Twist 1 could interact with p300 by coimmunoprecipitation. The transcriptional activity of FoxM1 promoter was further upregulated by co-transfection of p300 and Twist 1 (Figure 7E). Moreover, we further abolished p300 expression using siRNA oligos in NCI-N87 cells (Figure 7F). As expected, Twist 1-induced FoxM1 expression was significantly blunted by silence of p300 (Figure 7G-7H). The above data demonstrated that Twist 1 upregulated FoxM1 expression through a recruitment of transcriptional coactivator complexes, including p300.

## Discussion

In the current study, our results imply Twist 1 as a critical regulator of gastric cancer progression. This is suggested by several lines of evidence. First, Twist 1 overexpression promotes while its deficiency inhibits cell proliferation as shown by BrdU and cell-cycle analysis. Second, Twist 1 affects genes expression of cell-cycle modulators, including the CDK inhibitors p21Cip1, p27Kip1, the CDK regulator Cyclin B1, Cyclin D1, Cyclin D2 and Cyclin E. Third, Twist 1 directly upregulates FoxM1 expression at the transcriptional level, through recruitment of P300. Additionally, knockdown of endogenous FoxM1 expression attenuates the proliferative effect of Twist 1, suggesting that FoxM1 is essential for the roles of Twist 1 in gastric cancer cells. 

In adults, Twist 1 is mainly expressed in precursor cells including the osteoblastic, myogenic, odontoblastic and myelomonocytic lineages, maintaining their undifferentiated state [17]. Besides, Twist1 is also an important regulator of many other biological processes, including mesenchymal development and brown fat metabolism. For instance, Twist1 is believed to inhibit osteoblast differentiation by negatively regulating Runx2 [17]. Besides, Twist-1 is selectively expressed in adipose tissue, interacts with PGC-1α, to suppress mitochondrial metabolism and uncoupling. As a result, transgenic mice overexpressing Twist-1 in the adipose tissue are prone to high-fat-diet-induced obesity, whereas its heterozygous knockout mice are obesity resistant [18]. Moreover, Twist 1 nuclear expression was also observed in non-noncancerous epithelium, suggesting that Twist 1 may have a physiologic role in normal cells [19]. Subsequent studies demonstrate that Twist 1 expression is correlated with potent invasiveness as well as poor prognosis in epithelial cancer. In gastric cancer, a recent report has shown overexpression of the Twist 1 gene is more frequently found in diffuse-type carcinoma tissues with high N-cadherin gene expression [20]. However, no definitive results have indicated Twist promotes the proliferation of gastric cancer. Our findings suggested Twist probably promoted gastric cancer cell proliferation, using the BrdU incorporation assays. In addition, FoxM1 was firstly identified as a novel transcriptional target of Twist 1. 

FoxM1 binds promoter regions with a preference for a consensus [TAAACA] recognition sequence, although with lower affinity than other forkhead proteins [21]. Its expression is restricted to proliferating cells, and excluded from quiescent and terminally differentiated cells. It controls the expression of genes required for both G1/S and G2/M transition and is essential for mitotic entry and progression, ensuring the maintenance of chromosome stability [22,23]. Amplifications of FoxM1 gene has been reported in numerous tumors such as pancreatic carcinomas, breast cancer and hepatocellular carcinoma [24-27]. In gastric cancer, FoxM1 expression is also up-regulated and its inhibition leads to cellular senescence, which is at least in part, dependent on p27 kip1 [28,29]. In addition to its positive role on cell proliferation, FoxM1 has been shown to play roles in other cancer-related processes, such as invasion and metastasis [30]. Its expression levels correlate with poor prognosis and metastasis in different tumors including gastric carcinoma [31], suggesting the possibility of using FoxM1 as a prognosis and/or diagnosis marker. Taken together, FoxM1 is a promising and attractive target for cancer therapy. Therefore, it is critical to understand how FoxM1 is regulated in order to design better therapeutic approaches. 

FoxM1 expression is tightly regulated both at the mRNA and protein levels. Its abundance increases at the entry of S-phase, peaks during G2 and M, and is degraded during mitotic exit. Similarly, its transcriptional activity is tightly regulated throughout the cell cycle by multisite phosphorylation by different kinases, and its counteracting phosphatases, reaching its maximum activity in the G2 phase of the cell cycle [32]. Recently, it has been reported that FoxM1 expression can also be modulated by microRNAs. FoxM1 has been identified as a direct target of miR-134, whose levels are inversely correlated with the invasive potential of some NSCLC cells [33]. Moreover, FoxM1 is also repressed by miR-370 in gastric cancer cells [29], suggesting that further studies are required to investigate whether Twist 1 could cooperate these regulatory pathways. 

In conclusion, we here show that Twist 1 overexpression leads to a significant up-regulation of FoxM1, which plays a key role in cell cycle progression in gastric cancer cells. In contrast, knockdown of Twist 1 reduces FoxM1 expression, suggesting that FoxM1 might be a direct transcriptional target of Twist 1. We further reveal that Twist 1 could bind to the promoter region of FoxM1, and subsequently recruit p300 to induce FoxM1 mRNA transcription. Therefore, our results uncover a previous unknown Twist 1/FoxM1 regulatory pathway, which may help to understand the mechanisms of gastric cancer proliferation.

## Supporting Information

Figure S1 **(A-B) mRNA and protein levels of E-cadherin in NCI-N87 (A) and AGS (B) cells transfected with adenoviruses expressing empty vector (EV) or Twist 1.**
(TIF)

Figure S2 **(A-B) The cell proliferative potential (BrdU) was determined in NCI-N87 (A) or AGS (B) cells without or with transfection of empty vector (EV) or GFP siRNA.** (C) Endogenous Twist 1 expression was determined by western blot in four gastric cancer cells (NCI-N87, AGS, HGC-27 and MGC80-3). The growth curve of four cell lines was measured.
(TIF)

Figure S3 **(A-B) mRNA (A) and protein (B) levels of FoxM1 in NCI-N87 cells transfected with siRNA oligos against NCI-N87 or negative control (Ctrl).** (C) Cell proliferation activity was measured by BrdU assays in NCI-N87 cells. Cells were pre-transfected with siRNA oligos for 24 hours and then transfected with empty vector (EV) or Twist 1 for another 24 hours.
(D) mRNA levels of p21, p27, Cyclin B1, Cyclin D1, Cyclin D2 and Cyclin E were determined by real-time PCR in NCI-N87 cells. Cells were pre-transfected with siRNA oligos for 24 hours, and then transfected with empty vector (EV) or Twist 1 for another 24 hours.
(TIF)
