# Supplementary figures and images for: Twist1 Promotes Gastric Cancer Cell Proliferation through Up-Regulation of FoxM1
Source: PLoS One. 2013 Oct 24;8(10):e77625. doi: 10.1371/journal.pone.0077625 (PMC3812021; doi:10.1371/journal.pone.0077625)

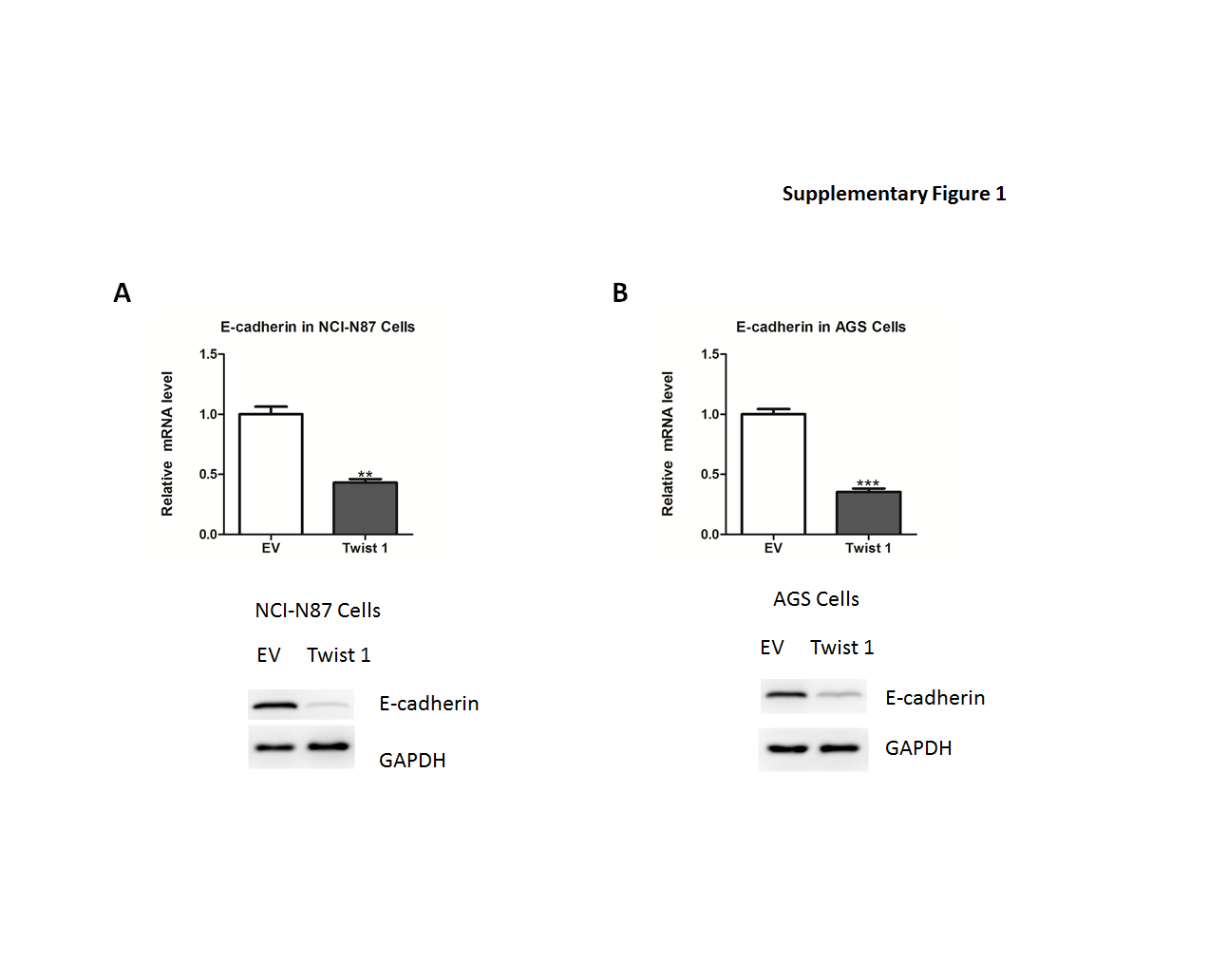

Supplement: Figure S1 — (A-B) mRNA and protein levels of E-cadherin in NCI-N87 (A) and AGS (B) cells transfected with adenoviruses expressing empty vector (EV) or Twist 1. (TIF) [file pone.0077625.s001.tif]

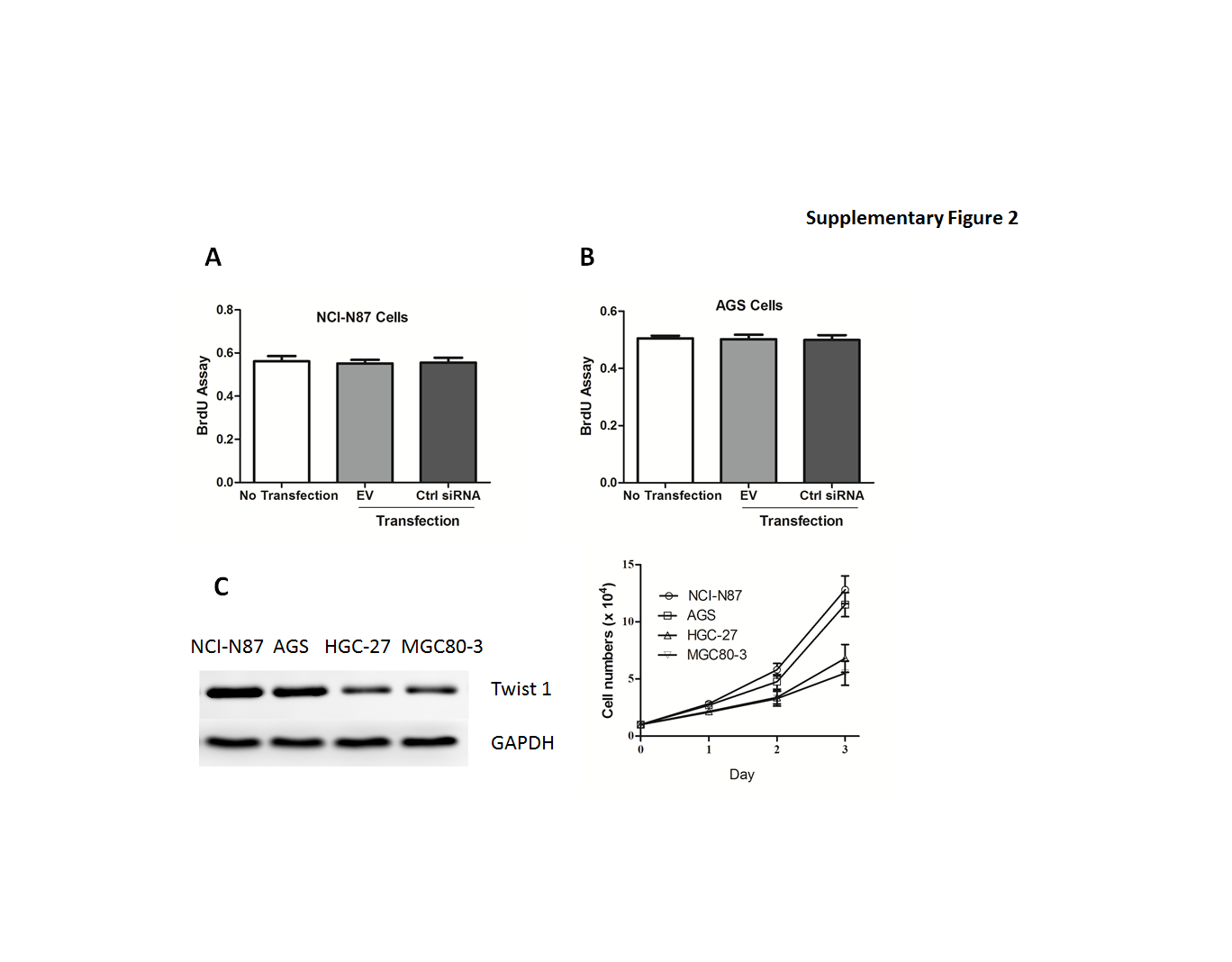

Supplement: Figure S2 — (A-B) The cell proliferative potential (BrdU) was determined in NCI-N87 (A) or AGS (B) cells without or with transfection of empty vector (EV) or GFP siRNA. (C) Endogenous Twist 1 expression was determined by western blot in four gastric cancer cells (NCI-N87, AGS, HGC-27 and MGC80-3). The growth curve of four cell lines was measured. (TIF) [file pone.0077625.s002.tif]

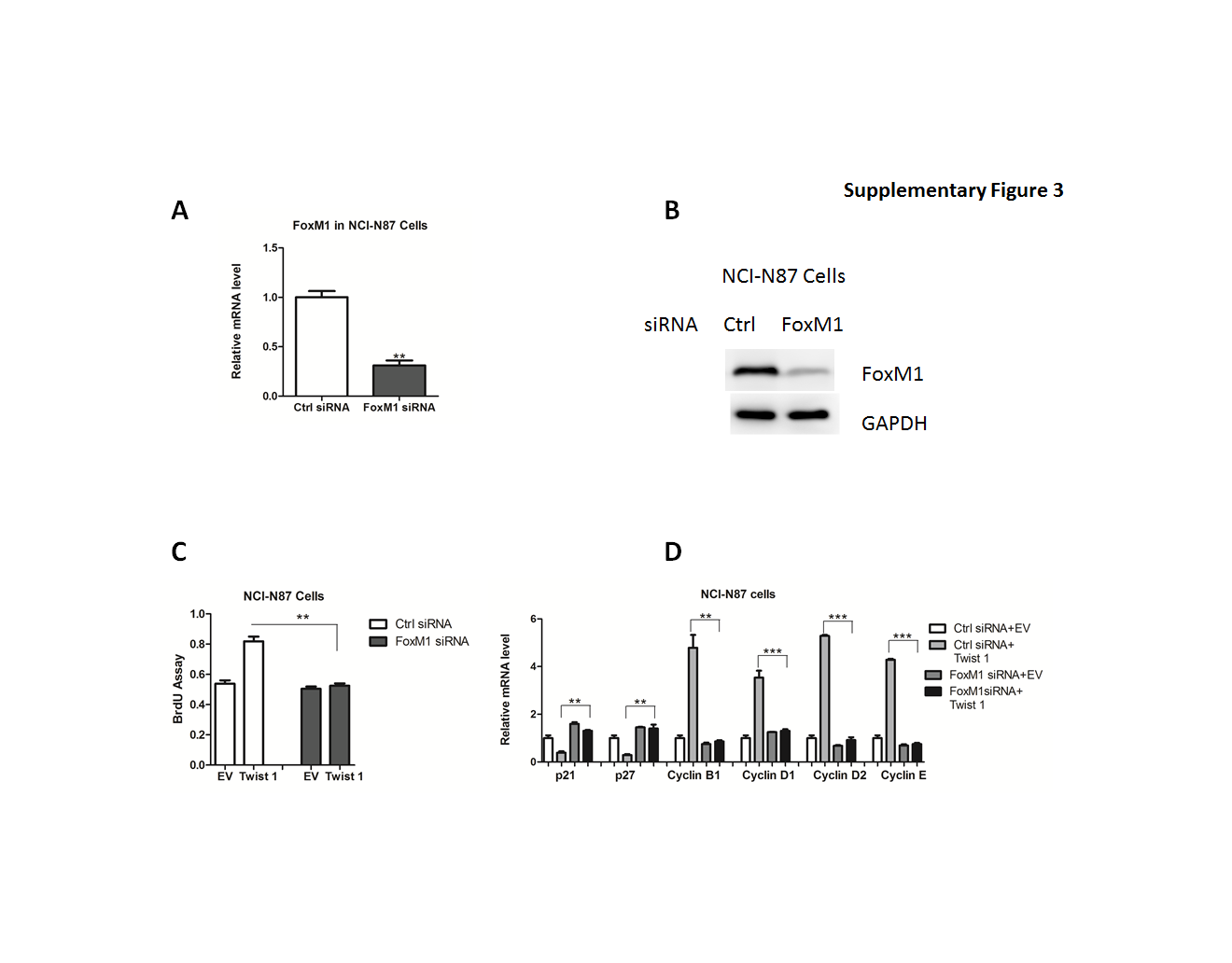

Supplement: Figure S3 — (A-B) mRNA (A) and protein (B) levels of FoxM1 in NCI-N87 cells transfected with siRNA oligos against NCI-N87 or negative control (Ctrl). (C) Cell proliferation activity was measured by BrdU assays in NCI-N87 cells. Cells were pre-transfected with siRNA oligos for 24 hours and then transfected with empty vector (EV) or Twist 1 for another 24 hours. (D) mRNA levels of p21, p27, Cyclin B1, Cyclin D1, Cyclin D2 and Cyclin E were determined by real-time PCR in NCI-N87 cells. Cells were pre-transfected with siRNA oligos for 24 hours, and then transfected with empty vector (EV) or Twist 1 for another 24 hours. (TIF) [file pone.0077625.s003.tif]
